# N-terminal pyroglutamate formation in CX3CL1 is essential for its full biologic activity

**DOI:** 10.1042/BSR20170712

**Published:** 2017-08-24

**Authors:** Astrid Kehlen, Monique Haegele, Livia Böhme, Holger Cynis, Torsten Hoffmann, Hans-Ulrich Demuth

**Affiliations:** 1 Probiodrug AG, Weinbergweg 22, Halle (Saale) 06120, Germany; 2 Institute of Medical Microbiology, Medical School, Martin-Luther-University Halle-Wittenberg, Weinbergweg 22, Halle (Saale) 06120, Germany; 3 Department of Drug Design and Target Validation, Fraunhofer Institute for Cell Therapy and Immunology, Weinbergweg 22, Halle 06120, Germany; 4 Deanary of Research, Medical Faculty, Martin-Luther-University Halle-Wittenberg, Magdeburger Str. 8, Halle (Saale) 06112, Germany

**Keywords:** CCL2, fractalkine, glutaminyl cyclase, gene expression, ICAM1/CD54

## Abstract

CX3CL1 (fractalkine) is a unique member of the CX3C chemokine family and mediates both adhesion and cell migration in inflammatory processes. Frequently, the activity of chemokines depends on a modified N-terminus as described for the N-terminus of CCL2 modified to a pGlu- (pyroglutamate) residue by QC (glutaminyl cyclase) activity. Here, we assess the role of the pGlu-modified residue of the CX3CL1 chemokine domain in human endothelial and smooth muscle cells. For the first time, we demonstrated using MS that QC (*QPCT*, gene name of QC) or its isoenzyme isoQC (iso-glutaminyl cyclase) (*QPCTL*, gene name of isoQC) catalyse the formation of N-terminal-modified pGlu-CX3CL1. Expression of *QPCT* is co-regulated with its substrates *CCL2* and *CX3CL1* in HUVECs (human umbilical vein endothelial cells) and HCASMCs (human coronary artery smooth muscle cells) upon stimulation with TNF-α and IL-1β whereas *QPCTL* expression is not affected. By contrast, inhibition of the NF-κB pathway using an IKK2 inhibitor decreased the expression of the co-regulated targets *QPCT, CCL2*, and *CX3CL1*. Furthermore, RNAi-mediated inhibition of *QPCT* expression resulted in a reduction in *CCL2* and *CX3CL1* mRNA. In HCASMCs, N-terminal-modified pGlu1-CX3CL1 induced a significant stronger effect on phosphorylation of ERK (extracellular signal regulated kinase) 1/2, Akt (protein kinase B), and p38 (p38 mitogen-activated protein kinase) kinases than the immature Gln1-CX3CL1 in a time- and concentration-dependent manner. Furthermore, pGlu1-CX3CL1 affected the expression of *CCL2, CX3CL1*, and the adhesion molecule *ICAM1/CD54* (intercellular adhesion molecule-1) inducing in higher expression level compared with its Gln1-variant in both HCASMCs and HUVECs. These results strongly suggest that QC-catalysed N-terminal pGlu formation of CX3CL1 is important for the stability or the interaction with its receptor and opens new insights into the function of QC in inflammation.

## Introduction

Increased chemokine levels and monocyte activation are common components of the pathogenesis of inflammatory diseases including atherosclerosis and chronic lung diseases. Especially the chemokines CCL2 (MCP-1, monocyte chemoattractant protein-1) and CX3CL1 (fractalkine) are described as key players for the attraction and migration of monocytes into the inflamed tissue. In contrast with the secreted CCL2, CX3CL1 is synthesized as a transmembrane protein with its chemokine domain presented on an extended highly glycosylated mucin-like stalk [1,2]. The membrane-bound CX3CL1 promotes the integrin-independent adhesion by binding with its G-protein-coupled, 7-transmembrane receptor CX3CR1 (fractalkine receptor 1) [3] and is a survival signal for circulating monocytes [4]. Soluble CX3CL1 can be cleaved from the cell surface expressed CX3CL1 by metalloproteinases such as ADAM10, ADAM17, or MMP2 [5–8]. In contrast with the membrane-tethered chemokine, soluble CX3CL1 can act as a classical diffusible chemoattractant and can promote the formation of transepithelial dendrites [4,9]. Collectively in the periphery, CX3CL1/CX3CR1 interactions seem to play a critical role in inflammation both affecting leucocyte recruitment and local intercellular communication [4]. Interestingly, the N-terminus of both chemokines CCL2 and CX3CL1 possess a glutamine in the first position and could be post-translationally modified by QCs to form a pGlu- (pyroglutamate) residue. Recently, we and others have shown that the chemotactic activity of CCL2 depends on a modified N-terminus of the polypeptide, particularly, the formation of a pGlu-residue protecting against proteolytic degradation *in vivo* [10,11]. The formation of N-terminal pGlu-residue is an important maturation step during synthesis and secretion of not only CCL2 but also of hormones such as thyrotropin-releasing hormone (TRH) and gonadotropin-releasing hormone (GnRH) [12–14]. The N-terminal pGlu of CCL2 can be formed by both QC (E.C. 2.3.2.5) and its isoenzyme, the isoQC (isoQC) [10,15]. Two distinct genes termed as *QPCT* (gene name of QC) (QC; NM_012413) and *QPCTL* (gene name of isoQC) (isoQC; NM_017659) are coding for different proteins with QC activity. In contrast with the secreted QC, isoQC is exclusively localized within the Golgi complex. IsoQC shows 46% sequence identity with the QC, and exhibits nearly identical substrate specificity *in vitro* [10,16].

Here, we describe CX3CL1 as a substrate for both the enzymes QC and isoQC. We further demonstrate that under inflammatory conditions, a co-regulation of the substrates CCL2 and CX3CL1 with their modifying enzyme QC in HUVECs (human umbilical vein endothelial cells) and HCASMCs (human coronary artery smooth muscle cells). Importantly, signalling of CX3CL1 depends on its modified N-terminus for activation of ERK (extracellular signal regulated kinase) 1/2, p38 (p38 mitogen-activated protein kinase), and Akt (protein kinase B) kinase as well as for induction of CCL2 and the adhesion molecule ICAM1/CD54 (intercellular adhesion molecule-1) in HUVECs and HCASMCs. Our results further support a role of QC in inflammatory processes.

## Methods

Human QC (EC 2.3.2.5) and its isoenzyme isoQC were expressed and purified as previously described [16].

For stimulation assays, recombinant human CX3CL1 chemokine domain (#300-31, PeproTech, Hamburg, Germany) was solubilized in aqua dest. (100 µg/ml). To generate the N-terminal pGlu modification (pGlu1-CX3CL1), CX3CL1 (Gln1-CX3CL1; 1 mg/ml) was diluted 1:10 in PBS and incubated with human recombinant QC (6 µg/ml) at 37°C for 2 h. Both CX3CL1 forms were aliquoted and stored at ≤ –20°C until use. The pGlu-CX3CL1 was used without further depletion of QC.

Antibodies were purchased from Cell Signalling (Frankfurt am Main, Germany). The following antibodies were used: anti-pERK (p-p44/42 MAPK (Erk1/2, Thr^202^/Tyr^204^, 20G11, rabbit mAb, #4376), anti-pP38 (p-p38 MAPK, Thr^180^/Tyr^182^, 12F8, rabbit mAb, #4631), anti-pAkt^Ser473^ (p-Akt (Ser^473^), D9E, XP® rabbit mAb, #4060), anti-ERK (p44/42 MAPK (Erk1/2), 137F5, rabbit mAb, #4695), anti-P38 (p38 MAPK antibody, #9212) and anti-Akt (Akt (pan), C67E7, rabbit mAb, #4691) and as secondary antibody, goat anti-rabbit-IgG (HRP–conjugated, #7074) was used.

Pooled *QPCT*-siRNA (ON-TARGETplus SMARTpool QPCT) and non-target control (NTC) were obtained from Dharmacon (Thermo Fisher Scientific, Karlsruhe, Germany), pool of four single FlexiTube siRNAs were obtained from Qiagen (Hilden, Germany; for sequences see Supplementary Table S1).

### MALDI-TOF MS

A 25 µM CX3CL1 solution was prepared in Tris buffer (20 mM, pH 8.0 adjusted with HCl), mixed to a final concentration of 10 µM with Tris buffer and 0.7 µg/ml of the enzymes QC or isoQC and incubated at 37°C. After adding the enzyme, several samples were taken at indicated time points. The cyclization of N-terminal glutamine residue was stopped with equal amounts of 0.1% trifluoroacetic acid. Afterwards, samples were purified with ZipTip C18; Merck Millipore, Darmstadt, Germany) according to the instructor’s manual and mixed with the matrices α-cyano-4-hydroxycinnamic acid (MW: 500–3000 g/mol), ferulic acid (MW: 3000–4000 g/mol) or sinapic acid (3,5-Dimethoxy-4-hydroxycinnamic acid, MW: >4000 g/mol; all Sigma–Aldrich, Taufkirchen, Germany) at a ratio of 1:1. In the case of cyclization of CX3CL1 in human serum, serum was diluted 1:10 with Tris buffer. MALDI-MS was performed using the Voyager DE Pro (Applied Biosystems, Darmstadt, Germany) in a linear mode. One microlitre of the analyte/matrix mixture was spotted on to the sample plate and air dried. The analytes were ionized by a nitrogen laser pulse (337 nm) and accelerated under 20 kV with a time-delayed extraction before entering the TOF mass spectrometer. Detector operation was in the positive ion mode ranging from 2000 to 20000 amu (atomic mass unit). Insulin and myoglobin (Sigma–Aldrich) were used for calibration in this range according to the instructor’s manual from Applied Biosystems. Each spectrum represented the sum of at least 6 × 100 laser pulses.

### Cell culture and stimulation

Primary HCASMCs obtained from normal regions of human coronary arteries from two independent donors were obtained from PromoCell (Heidelberg, Germany) and cultured according to manufacturer’s instructions using SMC growth medium 2 with supplements (SMC-2, PromoCell, #C-22062). All experiments were performed following 24-h serum starvation in serum-free SMC-2 medium containing 0.1 % BSA (Sigma–Aldrich).

Cultivation was done in EGM^TM^-2 medium with serum supplements and growth factors (Lonza, #CC-3162) following the manufacturer's instructions. For stimulation experiments, cells were seeded in 24-well plates and treated with TNF-α (#300-01A) and IL-1β (#200-01B) (each 10 ng/ml; PeproTech, Hamburg, Germany) for 24 h. Compounds IKK2 inhibitor IV (#401481) and U0126 MEK inhibitor (#662005) (both Calbiochem) were added 30 min before cytokine treatment. For signalling experiments, stimulation with 300 ng/ml Gln1-CX3CL1 or pGlu1-CX3CL1 for the indicated time intervals occurred in serum-free medium containing 0.1% BSA. For RNAi studies, HUVECs were transfected with siRNA pools or NTC (each 100 nM) according to manufacturer’s instructions using transfection reagent DharmaFECT 1 (Dharmacon, #T-2001). Until stated otherwise, independent replicates for the experiments were done at different days with different cell preparations or different passages of the same preparation (passages: 2–11 for HUVECs, passages: 3–8 for HCASMCs). Details including number of replicates are given in the figures legends.

### Western blotting

Protein lysates (20 µg) were separated on a 4–12 % NuPage Bis-Tris gel (Life Technologies, Darmstadt, Germany) and transferred on to nitrocellulose membrane (Roti-NC, 0.2 µm, Roth, Karlsruhe, Germany). Blots were blocked with blocking buffer (5% w/v dried milk (Roth)/TBS/0.1% Tween-20 (TBS-T)) for 60 min. Blots were incubated with primary antibody in blocking buffer overnight at 4°C, then incubated for 60 min at room temperature with secondary antibodies which were detected using the SuperSignal West Femto Kit (Thermo Fisher Scientific, #34095) and CCD-Imagingsysteme FUSION-FX7 (Peqlab, Erlangen, Germany). Western blots were stripped for 5 min at 37°C and 15 min at room temperature in Restore Western Blot Stripping Buffer (Thermo Fisher Scientific, #21059).

### Quantitative PCR

RNA was isolated using the NucleoSpin RNA II Kit (Macherey Nagel, Düren, Germany) according to the manufacturer’s instructions and RNA concentration was measured using a NanoDrop 2000 spectrophotometer (Peqlab, Erlangen, Germany). RNA was reverse transcribed into cDNA using random primers (Roche, Mannheim, Germany), dNTPs (Peqlab) and Superscript III (Life Technologies). qPCR (quantitative PCR) was performed in the Rotor-Gene RG3000 (Corbett Research, Sydney, Australia) using the Rotor-Gene SYBR Green Mastermix (Qiagen, Hilden, Germany) according to the manufacturer’s instructions and the QuantiTect primer assays Hs_QPCT_1_SG (NM_012413) and QPCTL_1_SG (NM_017659) (Qiagen) or specific primers for *CCL2* (NM_002982.3), *ICAM1/CD54* (NM_000201.1), *CX3CL1* (NM_002996.3), *GAPDH* (NM_002046.3), *YWHAZ* (NM_003406.2) all synthesized by Metabion (Martinsried, Germany). The primer sequences are summarized in Supplementary Table S2. Relative amounts of gene expression were determined with the Rotor-Gene software version 6.1 using the comparative quantification method. *GAPDH* and *YWHAZ* were used as reference genes. Melt-curve analysis following PCR showed a single product for all the amplicons.

### Immunoassays

For determination of human total-CCL2, a specific ELISA was used as described previously [10]. CX3CL1 and soluble ICAM1/CD54 (sICAM1) concentrations in conditioned medium were determined using the MILLIPLEX MAP kit (Merck Millipore) and the analysing system Bio-Plex 200 (Bio–Rad, Munich, Germany). Calibration of the assay occurred via Bio-Plex Calibration Kit in specific Bio-Plex MCV plates (Bio–Rad). After incubation of plates at 4°C overnight, analytes were measured and concentrations were calculated using the Bio-Plex-Manager software (version 4.1.1).

### ICAM1/CD54 detection by flow cytometry

Cells were detached with accutase (PAA, Cölbe, Germany), washed with PBS buffer and resuspended in FACS staining buffer (PBS, 5% FCS, 2 mM EDTA). After blocking with FcR blocking reagent (Miltenyi Biotec, Bergisch Gladbach, Germany, #130-059-901), cells were stained with the APC–conjugated mouse anti-human CD54 antibody (BD Bioscience, Heidelberg, Germany, #559771) or the isotype control antibody (eBioscience, Frankfurt am Main, Germany, #48-4301-80) for 30 min, at 4°C in the dark. Flow cytometry was performed using the FACS Calibur instrument with CellQest software (BD Bioscience).

### QC activity determination

After stimulation or siRNA transfection, conditioned medium was concentrated to a tenth of the original volume (Vivaspin 6 columns 10000 MWCO; Sartorius, Göttingen, Germany). Cells were pelletized, sonicated in buffer (10 mM Tris, 100 mM NaCl, 5 mM EDTA, 0.5% Triton X-100, 10% glycerol, pH 7.5) and centrifuged at 16000×***g*** for 30 min and 4°C. The protein concentration of the resulting supernatant and the medium concentrate was determined using the method of Bradford (RotiQuant, Roth). The QC/isoQC activity was measured applying HPLC assay as described previously [35,36].

### Statistical analysis

All quantificative data are presented as mean ± S.D. Differences between experimental groups were evaluated for significance using Student’s *t* test for unpaired data or one-way ANOVA when appropriate by SigmaPlot software (Systat, Erkrath, Germany).

## Results

### CX3CL1 is a substrate of QC

**Figure 1:**
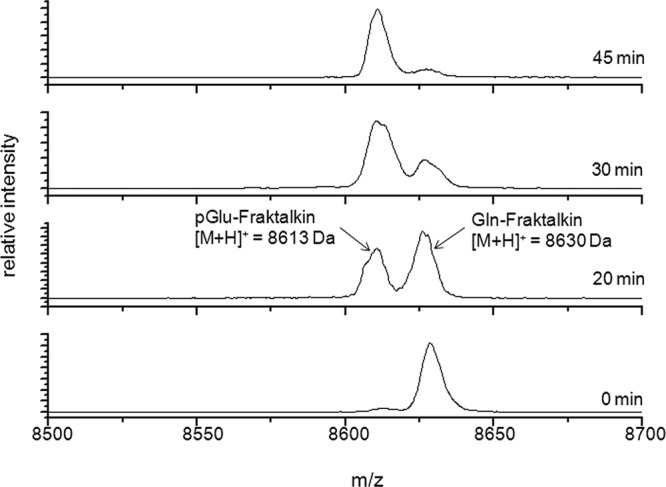
CX3CL1 is a substrate of human QC After 0, 20, 30 and 45 min of incubation at 37°C, N-terminal modification of Gln1-CX3CL1 by human recombinant QC was monitored using MALDI-TOF-MS. The product peak with a mass of 8613 Da resulted from a loss of ammonia during cyclization reaction (minus 17 Da) and exceeds the peak of the original mass of 8630 Da after 30 min of incubation with QC.

The N-terminus of the chemokine CX3CL1 has a glutamine in the first position. To confirm whether QC and isoQC can catalyse N-terminal pGlu formation, the recombinant chemokine domain of CX3CL1 was incubated with QC or isoQC and the resulting reaction products were analysed by MS. Thirty minutes after adding the enzymes the peak of pGlu1-CX3CL1 had a much higher intensity than the peak in the non-modified form Gln1-CX3CL1 (Figure 1 shows spectra with QC). After 45 min, the reaction was almost finished. Only a small signal of the original mass could be detected at this time point. Due to its high mass (theoretical mass of Gln1-CX3CL1: 8635 Da and of pGlu1-CX3CL1: 8618 Da) it comes to a mass drift of CX3CL1 during the MALDI-TOF-MS measurements resulting in a deviant mass of 8630 and 8613 Da for Gln1-CX3CL1 and pGlu1-CX3CL1 respectively. This deviation of 0.058% is near the mass accuracy of 0.05% specified by the manufacturer for the used equipment (Voyager DE Pro, linear mode, external calibration). However, the differences between theoretical and observed values were always constant. Similar results for the conversion of immature Gln1-CX3CL1 to pGlu1-CX3CL1 were seen with recombinant human isoQC (results not shown) or human serum as matrix (Supplementary Figure S1). To summarize, we could show for the first time that human CX3CL1 is a substrate for both QC and isoQC. Both enzymes are capable of catalysing the post-translational formation of an N-terminal pGlu-residue.

### Co-regulation of QC and its substrates CCL2 and CX3CL1 upon stimulation with TNF-α/IL-1β

**Figure 2:**
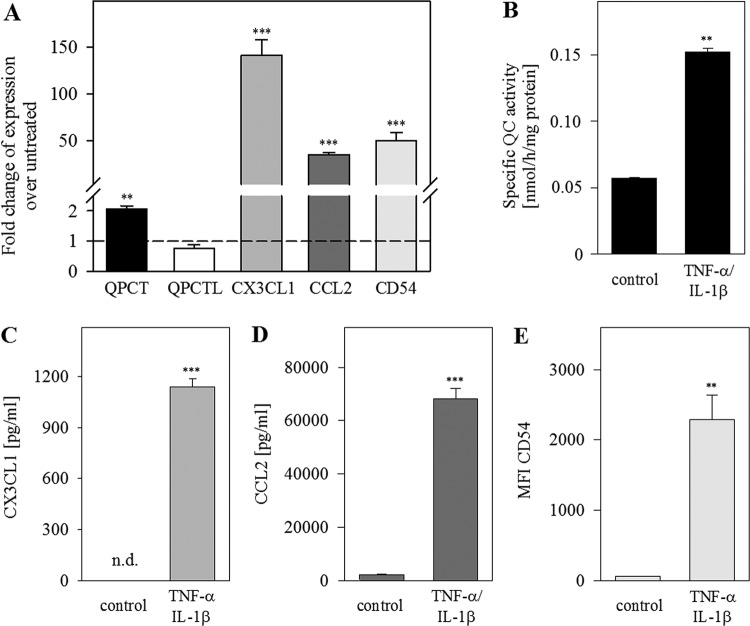
Co-regulation of QC and its substrates CCL2 and CX3CL1 upon stimulation with TNF-α/IL-1β in HUVECs HUVECs were treated with TNF-α/IL-1β (10 ng/ml) for 24 h. (**A**) qPCR analysis was performed for the indicated genes. (**B**) Specific QC activity in conditioned medium was measured by HPLC and normalized to protein concentration. The basal activity of the culture medium without cells was subtracted. (**C**) CX3CL1 was quantified via immunoassay (MILLIPLEX MAP). (**D**) CCL2 concentration in supernatant was measured with specific human CCL2 ELISA. (**E**) Surface expression of CD54 was analysed by flow cytometry. Data from three independent experiments on different days are presented as mean ± S.D. (**P<0.01, ***P<0.001. Abbreviation: n.d., not detectable).

To study the regulation of QC and its chemokine substrates, primary endothelial cells (HUVECs) were treated with the proinflammatory cytokines TNF-α/IL-1β. Using qPCR, ELISA and flow cytometry for measuring gene and protein expression, we detected an increase in CX3CL1, CCL2 and ICAM1/CD54 levels (Figure 2A,C–E). Furthermore, we found an increase in *QPCT* mRNA expression up to two-fold (*P*<0.01), whereas *QPCTL* gene expression was not changed (Figure 2A). A three-fold increase in specific QC activity was measured in conditioned medium of stimulated cells (*P*<0.01, Figure 2B), but no change in QC/isoQC activity in cell extracts could be detected. This result is also indicative for the stimulation of the secreted QC but not of the Golgi resident isoQC.

**Figure 3:**
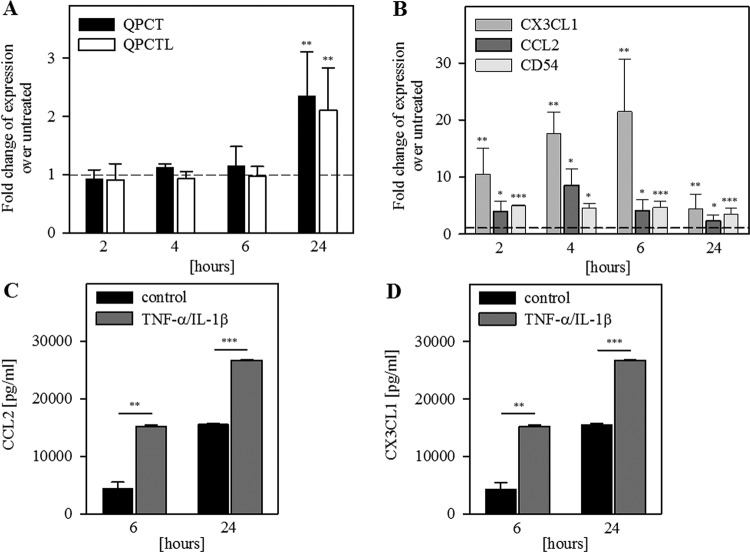
Co-regulation of QC and its substrates CCL2 and CX3CL1 in HCASMCs is time dependent HCASMCs were serum-starved for 24 h and then treated with TNF-α/IL-1β (10 ng/ml) for the indicated times. qPCR was performed for the indicated genes (**A**) *QPCT, QPCTL*; (**B**) *CX3CL1, CCL2, ICAM1/CD54*. Data from eight independent experiments (cells from two different donors) are expressed as fold-change over untreated and shown as mean ± S.D. (**C**) CCL2 was detected with immunoassay (MILLIPLEX MAP); (**D**) CX3CL1 was quantified with immunoassay (MILLIPLEX MAP); Data from three experiments performed in duplicates are shown as mean ± S.D. (*P< 0.05, **P<0.01, ***P<0.001).

To confirm the co-regulation of QC and its substrates, we studied the gene expression in a time-dependent manner in HCASMCs. Whereas the increase in *CX3CL1, CCL2* and *ICAM1/CD54* levels was detectable already 2 h after stimulation, *QPCT* and *QPCTL* transcripts were found to be increased only after 24 h (Figure 3).

In conclusion, we demonstrated a co-regulation of QC and its substrates CX3CL1 and CCL2, as well as of the adhesion molecule ICAM1/CD54 in both primary endothelial and smooth muscle cells.

### NF-κB-dependent regulation of QPCT and its substrates CX3CL1 and CCL2

**Figure 4:**
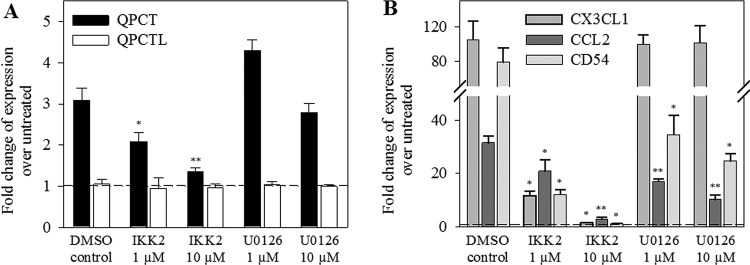
NF-κB-dependent co-regulation of *QPCT* and substrates *CCL2* and *CX3CL1* mRNA expression in HUVECs After treatment with the appropriate inhibitor IKK2 or U0126 (each 10 µM) for 30 min, HUVECs were cultured for 6 h with cytokines TNF-α/IL-1β (100 ng/ml). Results of qPCR are shown relative to the basal control levels (**A**) *QPCT, QPCTL*; (**B**) *CX3CL1, CCL2, ICAM1/CD54*. Data from three independent experiments are shown as mean ± S.D. (*P<0.05, **P<0.01, ***P<0.001).

NF-κB signalling is involved in rapid response to various stimuli, such as the cytokines TNF-α, IL-1β or infections, shear or oxidative stress. To assess whether NF-κB is involved in the regulation of QPCT and its substrates, we treated TNF-α/IL-1β-stimulated HUVECs with the inhibitor of the NF-κB pathway, IKK2 compound IV or with the ERK/MAPK inhibitor U0126. The IKK2 inhibitor was able to abolish the TNF-α/IL-1β-induced gene expression of *QPCT* and its substrates *CX3CL1* and *CCL2* as well as of *ICAM1/CD54* in a concentration-dependent manner (Figure 4). The ERK/MAPK inhibitor did not affect the *QPCT* and *CX3CL1* transcript levels, but did significantly reduced the *CCL2* as well as the *ICAM1/CD54* gene expression.

### QPCT-gene silencing reduces CX3CL1, CCL2 and CD54 expression

**Figure 5:**
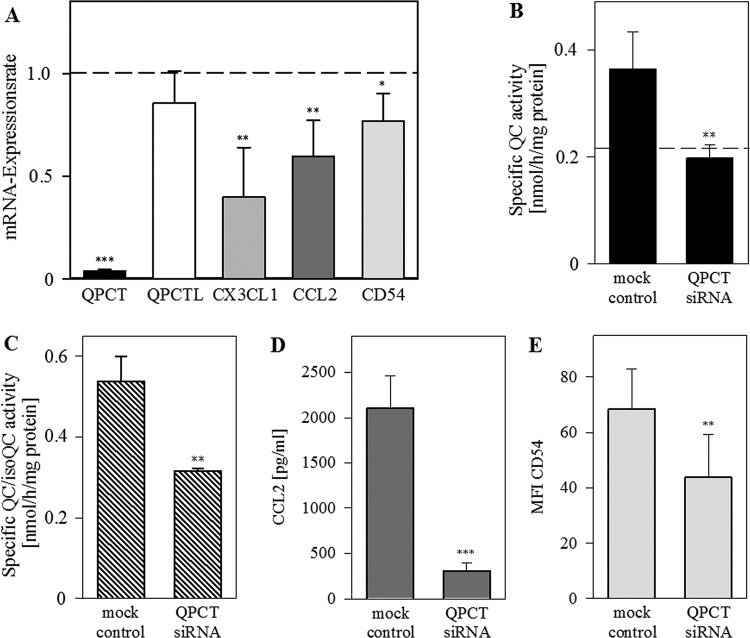
Transfection of HUVECs with QPCT siRNA resulted in down-regulation of substrates CX3CL1 and CCL2 HUVECs were treated with siRNA for over 72 h. (**A**) qPCR was performed for the indicated genes. (**B**) Specific QC activity in conditioned medium was measured by HPLC and normalized to protein concentration. The activity of basal medium without cells is presented as a horizontal line. (**C**) Specific QC/isoQC activity in cell extracts was measured by HPLC and normalized to protein concentration. (**D**) CCL2 was measured via hCCL2 ELISA. (**E**) ICAM1/CD54 was quantified via immunoassay (MILLIPLEX MAP). Data from six independent experiments are presented as mean ± S.D. (*P<0.05, **P<0.01, ***P<0.001).

Next, we studied the effect of QC knockdown on the expression of its substrates. We transfected HUVECs with *QPCT*-siRNA pools or with a NTC for 72 h. As shown in Figure 5A, *QPCT*-siRNA knockdown (reduction up to 3–5%; *P*<0.001) resulted in lowered mRNA amounts of *CX3CL1* (reduction up to 40% of control, *P*<0.01), *CCL2* (60% of control, *P*<0.01) and *ICAM1/CD54* (73% of control, *P*<0.05). Treatment of HUVECs with NTC had no significant impact on the expression of genes investigated. RNAi of *QPCT* decreased QC activity in conditioned medium of transfected cells to the basal level of the culture medium, which contained activity from the serum supplement (horizontal line in Figure 5B). In cell extracts of siRNA-transfected HUVECs, we found a significant reduction in QC/isoQC activity compared with mock treated cells (*P*<0.01, Figure 5C). The remaining enzyme activity certainly originated from the isoQC, which is located in the Golgi complex and was not affected by *QPCT*-siRNA transfection. Comparable with the decreased mRNA expression, protein levels of CCL2 and CD54 declined as well (Figure 5D,E).

### pGlu1-CX3CL1 induces ERK, p38 and Akt phosphorylation

**Figure 6:**
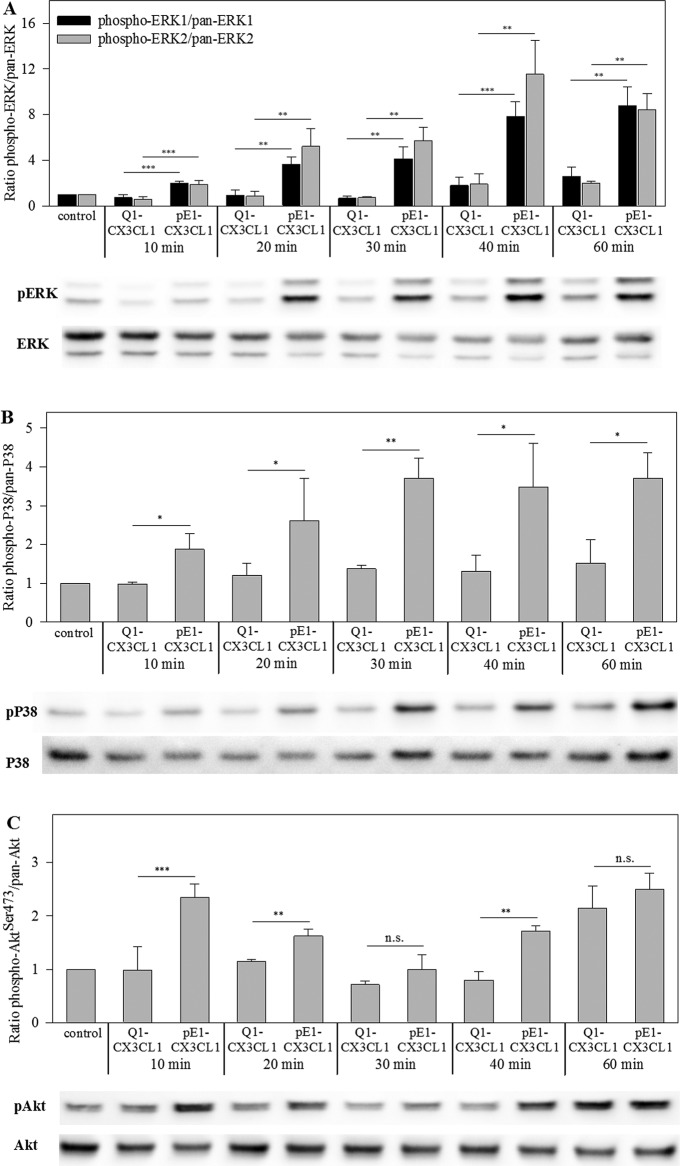
pGlu1-residue of CX3CL1 is important for phosphorylation of ERK1/2, Akt and p38 in HCASMCs HCASMCs were serum-starved for 24 h prior to treatment with Gln1-CX3CL1 or pGlu1-CX3CL1 (600 ng/ml) for the indicated times. Cell lysates were prepared and Western blotted. Membranes were stained with phosphospecific antibodies before stripping and reprobing with pan-specific antibodies. Quantification of the bands was done using FusionSoftware. Data from three independent experiments are shown as mean ± S.D. (*P<0.05, **P<0.01, ***P<0.001. Abbreviation: n.s., non-significance). One blot is shown representatively. (**A**) Ratio of p-ERK1/2/pan ERK1/2; (**B**) ratio of p-p38/pan p38; (**C**) ratio of pAkt^Ser473^/total Akt.

White et al. [17] published that mitogenic and anti-apoptotic effects of CX3CL1 require ERK and PI3K/Akt signalling in HCASMCs. Therefore, we studied the physiological relevance of pGlu1 formation on CX3CL1 for the activation of ERK, p38 and Akt signal transduction pathways. First, we confirmed a marginal CX3CR1 cell surface expression in HCASMCs and HUVECs by flow cytometry. Only pGlu1-CX3CL1 not Gln1-CX3CL1 was able to induce phosphorylation of kinases ERK1/2, p38 and Akt (Figure 6A–C). Phosphorylation of ERK1/2 and p38 started within 10 min and peaked between 40 and 60 min after induction. On the contrary, activation of Akt showed two peaks at 10 and 60 min. At any time, pGlu1-CX3CL1 induced a stronger phosphorylation of ERK1/2, p38 and Akt compared with Gln1-CX3CL1, which only activated the kinases at 40 and 60 min slightly. This indicates an important functional relevance of the pGlu-residue of CX3CL1 for induction of signalling in HCASMCs.

### pGlu1-CX3CL1 induces expression of CX3CL1, CCL2 and CD54

**Figure 7:**
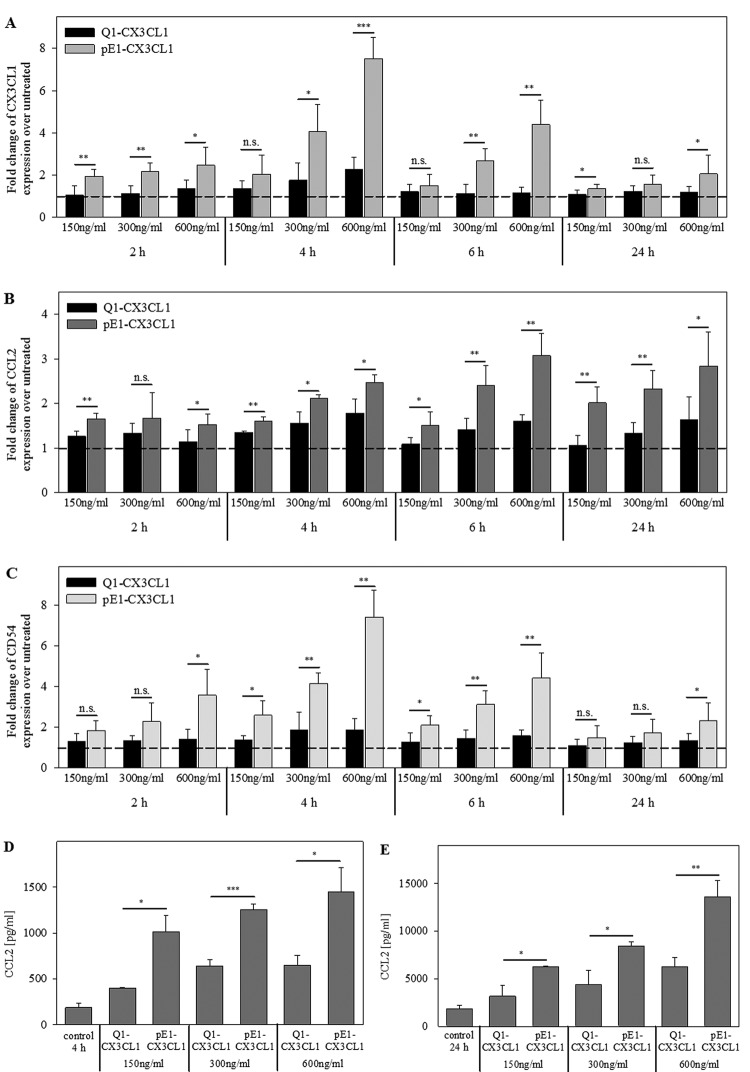
Induction of mRNA and protein expression in HCASMCs by CX3CL1: pGlu1-residue of CX3CL1 is important for induced expression of CX3CL1, CCL2 and ICAM1/CD54 HCASMCs were serum-starved for 24 h prior to treatment with Gln1-CX3CL1 or pGlu1-CX3CL1 for the indicated concentrations and times. qPCR was performed for the indicated genes (**A**) *CX3CL1*; (**B**) *CCL2*; (**C**) *ICAM1/CD54*. Data from five independent experiments (cells from two different donors) are expressed as fold-change over untreated. CCL2 levels were quantified with a specific hCCL2 ELISA 4 h (**D**) and 24 h (**E**) after treatment. Data from three independent experiments are shown as mean ± S.D. (*P<0.05, **P<0.01, ***P<0.001).

To prove the effect on gene expression as a subsequent result of CX3CR1 activation, HCASMCs were treated with Gln1-CX3CL1 or pGlu1-CX3CL1 in time- and concentration-dependent assays. Using qPCR, the transcript levels of *CX3CL1, CCL2, ICAM1/CD54, QPCT*, and *QPCTL* were analysed. pGlu1-CX3CL1 was more effective in induction of gene expression than Gln1-CX3CL1. Gln1-CX3CL1 could only up-regulate the mRNA levels up to two-fold whereas pGlu1-CX3CL1 induced mRNA expression in a concentration-dependent manner up to 7.5-fold for *CX3CL1*, 3.1-fold for *CCL2* and 7.4-fold for *ICAM1/CD54* (Figure 7A–C). Noticeably, the mRNA expression of *CX3CL1* and *ICAM1/CD54* reached their maxima at 4 h, whereas that of *CCL2* peaked later. After 24 h of incubation, *QPCT* gene expression presented a slight increase in 1.5-fold upon stimulation with 600 ng/ml pGlu1-CX3CL1 but not with Gln1-CX3CL1 (*P*<0.05). To confirm the differential gene expression on protein level, CCL2 protein was measured via CCL2 ELISA in the culture supernatant of HCASMCs. After 4 and 24 h, pGlu1-CX3CL1 increased the CCL2 protein levels significantly in a concentration-dependent manner compared with Gln1-CX3CL1 (Figure 7D,E).

**Figure 8:**
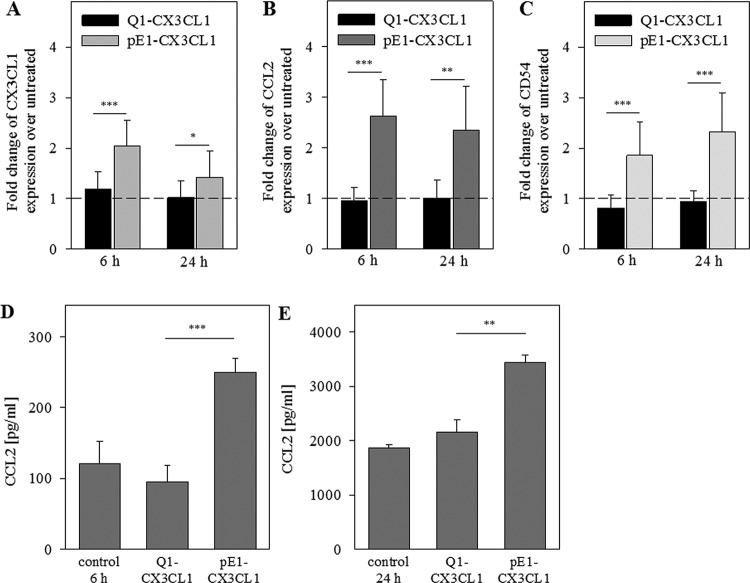
Induction of mRNA and protein expression in HUVECs by CX3CL1: N-terminal pGlu1-residue of CX3CL1 is important for induction of CX3CL1, CCL2 and ICAM1/CD54 expression HUVECs were treated with Gln1-CX3CL1 or pGlu1-CX3CL1 (300 ng/ml) for 6 and 24 h. (**A**–**C**) Results of qPCR are shown relative to the basal control levels. CCL2 concentrations in supernatant were quantified with a specific hCCL2 ELISA 6 h (**D**) and 24 h (**E**) after treatment. Data from four independent experiments (triplicates) are shown as mean ± S.D.

To further verify the importance of the N-terminal pGlu-residue for CX3CL1 activity, we also treated primary endothelial cells (HUVECs) with both CX3CL1 variants at a concentration of 300 ng/ml for 6 and 24 h. pGlu-CX3CL1 increased the transcript levels of *CX3CL1* up to 3.3-fold, of *CCL2* up to 2.6-fold and of *ICAM1/CD54* up to 2.4-fold, whereas Gln1-CX3CL1 did not affect transcript levels of these molecules at all (Figure 8A–C). Using ELISA, we quantified the CCL2 concentration after 6 and 24 h. Similar to HCASMC, the pGlu-form of CX3CL1 enhanced CCL2 protein concentrations in HUVEC supernatants 2.5-fold within 6 h (*P*<0.001) and 1.5-fold within 24 h (*P*<0.01) whereas Gln1-CX3CL1 shows no effect (Figure 8D,E). In conclusion, we demonstrated for the first time that the pGlu-residue on CX3CL1 is essential for CX3CR1 signalling and subsequent gene and protein expression in HCASMCs and HUVECs.

## Discussion

pGlu formation catalysed by QCs is an important post-translational step in maturation of chemokines as CCL2, CCL7, CCL8 and CCL13 protecting against N-terminal proteolytic degradation, e.g. by dipeptidyl peptidase 4/CD26 [10,11,18]. Here, we could identify the chemokine domain of CX3CL1 as an additional new QC substrate. The recombinant Gln1-CX3CL1 was converted into its pGlu-form by recombinant QC or isoQC as well as by QC activity of human serum. Interestingly, in the human serum assays, CX3CL1 was cleaved C-terminally resulting in a truncation of the peptide by the C-terminal RNG triplet (Supplementary Figure S1). This C-terminal cleavage of human CX3CL1 was independent of the N-terminal cyclization reaction.

Besides the protection of the N-terminus against exopeptidase degradation, it is well-known that the N-terminal pGlu-residue can help stabilize the proper conformation of proteins for binding to their receptors [12,14]. Recently, we reported that pGlu1-CCL2 is more effective in monocytic migration and CCR2-internalization assay compared with its immature Gln1 variant [10]. Here, we show the physiological relevance of pGlu1 formation on CX3CL1 for the activation of ERK, p38 and Akt signal transduction pathways in HASMCs. At any time point investigated, pGlu1-CX3CL1 induced a significant stronger effect on phosphorylation of ERK1/2 and p38 kinase than the Gln1-CX3CL1. Similar results were observed for Akt^Ser473^ phosphorylation at time points 10, 20 and 40 min upon chemokine stimulation. A similar time course of kinase phosphorylation by CX3CL1 was described for monocytic cells, CX3CR1-transfected HEK293T cells, neuroblastoma and osteoarthritis fibroblasts [19–23]. Ryu et al. [24] demonstrated in human aortic endothelial cells that CX3CL1 activates ERK1/2 phosphorylation and subsequently the VEGF-A/KDR-1-induced angiogenesis. Furthermore, in CX3CL1-stimulated human microvascular and umbilical endothelial cells changes in the phosphorylation status of ERK1/2 and JNK and alteration in the cytoskeleton were detected. Both processes are necessary for endothelial migration and angiogenesis in rheumatoid arthritis [23]. In rat aortic smooth muscle cells, CX3CL1 induced its own expression and was involved in cell–cell adhesion and cell proliferation via the PI3K/Akt^Thr308^/NF-κB pathway. Therefore, CX3CL1 was discussed as a mediator in initiation and progression of atherosclerotic vascular diseases [25]. Using HCASMCs, White et al. [17] showed that CX3CL1-induced anti-apoptosis is mediated by cross-talk to the epidermal growth factor receptor pathway. CX3CL1 induced shedding of epiregulin, which acted in an autocrine/paracrine manner to activate the epidermal growth factor receptor, leading to PI3K activation and Akt phosphorylation [17].

To study the translation of the pGlu1-CX3CL1-mediated signalling into the effects on differential gene expression, we quantified the RNA levels of *CCL2, ICAM1/CD54* and *CX3CL*1 itself. pGlu1-CX3CL1 induced higher amounts of *CCL2, ICAM1/CD54* and *CX3CL1* mRNA than the immature Gln1-CX3CL1 in both HUVECs and HCASMCs. In addition, we found significant higher CCL2 protein levels in the supernatant of HUVECs and HCASMCs upon pGlu1-CX3CL1 stimulation compared with the appropriate stimulation with Gln1-CX3CL1. These results strongly suggest that the QC-catalysed pGlu-CX3CL1 formation is required for an effective activation of the CX3CL1/CX3CR1 axis and the induction of molecules important for adhesion and migration as CCL2, ICAM1/CD54 and CX3CL1. The up-regulation of ICAM1/CD54 by CX3CL1 was also reported in mouse hearts *ex vivo* and in HUVECs via the Jak/Stat5 pathway [26]. In aortic smooth muscle cells, both increased transcription of mRNA and higher mRNA stability contributed to the CX3CL1-induced CX3CL1 expression [25]. In the monocyte/endothelial cell cross-talk, endothelial CX3CL1 potentiated monocytic CCL2 release that might contribute to the recruitment of monocytes into inflamed areas [27].

Recently, synergistic induction of CX3CL1 in response to the combined stimulation with TNF-α and IFN-γ was described for HUVECs [28] and human aortic smooth muscle cells [29]. Here, we demonstrated the co-regulation of the enzyme QC and its substrates CCL2 and CX3CL1 at the RNA and protein level upon stimulation with the proinflammatory cytokines TNF-α and IL-1β whereas the *QPCTL* gene expression was not affected. By contrast, inhibition of the NF-κB pathway using an IKK2 inhibitor decreased the expression of the co-regulated targets *QPCT, CCL2* and *CX3CL1*. Furthermore, RNAi-mediated inhibition of QPCT expression resulted in a reduction in *CCL2* as well as *CX3CL1* mRNA. Recently, we described a similar NF-κB-dependent co-regulation of QPCT and CCL2 in epithelial cells of thyroid carcinomas upon TNF-α/IL-1β treatment [30]. QC is believed to be involved in the pathology of several diseases like osteoporosis [31], melanoma [32], Alzheimer’s disease (AD) [33] and septic arthritis [34]. In an animal model of atherosclerosis (cuff-induced accelerated atherosclerosis in ApoE3*Leiden mice) inhibition of QC activity reduced the number of adhering monocytes, down-regulated CCL2 in the media and the intima, and reduced neointima formation and lumen stenosis [10]. Furthermore, especially for the nervous system there is growing evidence that CX3CL1 is an important regulator of microglia-neuron cross-talk [37] and an imbalance of this interaction may be an important part in the pathology of AD and other neurodegenerative diseases. In this context, the correlation found by Bridel et al. [38] in an AD biomarker study between QC activity and CSF levels of Aβ peptides, adhesion molecules (ICAM1, VCAM1) as well as members of the VEGF pathway (VEGFD and Flt1) is remarkable. Co-regulation of these molecules may be at least partially NF-κB or CX3CL1 dependent [24].

## Conclusion

**Figure 9:**
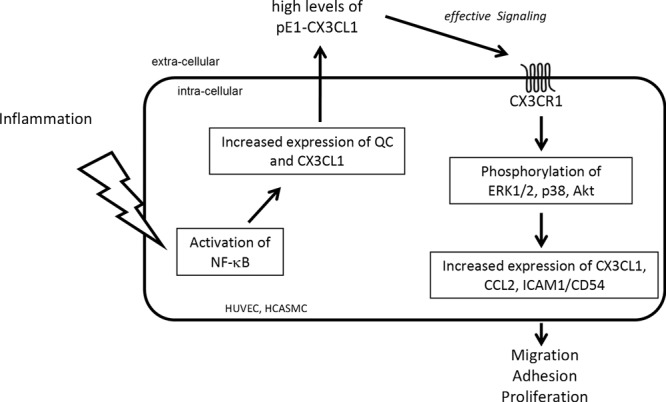
Role of pGlu1-CX3CL1 in HUVECs and HCASMCs Activated NF-κB increases the expression of QC and its substrate CX3CL1. pGlu formation catalysed by QC is an important post-translational step in CX3CL1 maturation. pGlu1-CX3CL1 can bind to the CX3CR1 expressed on the cell surface and activates the ERK1/2, p38 and Akt signal transduction pathways resulting in increased expression of chemokines and adhesion molecules as CX3CL1, CCL2 and ICAM1/CD54. Together, pGlu1-CX3CL1 functions as mediator and amplifier of cell migration, adhesion and proliferation.

Post-translational modification of N-terminal glutamine to pGlu by QC/isoQC is used as a cellular strategy to fine-tune the activity of the chemokines CX3CL1 and CCL2. The data presented here provide evidence that in case of an inflammatory stimulus, QC is co-regulated with its chemokine substrates CX3CL1 and CCL2. Furthermore, we demonstrated that the QC-dependent formation of the N-terminal pGlu of CX3CL1 is required for full physiologic activity of this chemokine and is a prerequisite for an effective activation of signalling pathways resulting in an increase in expression of molecules, involved in inflammation, proliferation, cell migration and adhesion (Figure 9) like CCL2, ICAM1 and CXCL3 itself. So, under inflammatory conditions where release of these chemokines could increase the co-regulation of QC dramatically ensures that all these chemokines are released in their fully active N-terminally cyclized form. If the missing N-terminal pGlu results in an increased accessibility for N-terminal proteolytic degradation or directly affects receptor interaction or activity could not be answered by the available data and should be clarified in future experiments.

Nevertheless, the data demonstrate the importance of the N-terminal pyroglutamyl modification also for CX3CL1-dependent signalling processes, and support the idea that reduction in pGlu-chemokine levels by inhibition of QC activity might result in a reduced inflammatory phenotype in chronic and acute diseases and provide a possible new treatment strategy.

## Supporting information

**Supplemental Table 1.** Pooled siRNAs consisted of 4 single siRNAs with the following sequences

**Supplemental Table 2.** Primer sequences of the human genes

**Fig 1 Supplement:** Conversion of Q1-CX3CL1 to pE1-CX3CL1 in human serum At time point 0 min, the major peak corresponds to a mass of 8630, which is close to the theoretical molecular weight (8639) of Q1-CX3CL1. After incubating Q1-CX3CL1 with human serum for 30 min, the major peak corresponds to a mass of 8613, which is close to the theoretical molecular weight (8622) of pE1-CX3CL1. The doublet at 8286/8303 corresponds to a shortened peptide produced by limited proteolysis at the C-terminus (release of Cterminal RNG tripeptide). The C-terminal cleavage is independent of N-terminal cyclisation reaction.

## Abbreviations

Aβ: beta amyloid
AD: Alzheimer's disease
ADAM: a desintegrin and metalloproteinase domain-containing protein
Akt: protein kinase B
APC: allophycocyanin
CCL2/MCP-1: CC-chemokine ligand-2 / monocyte chemoattractant protein-1
CSF: cerebrospinal fluid
CX3CL1: fractalkine
CX3CR1: fractalkine receptor 1
ERK: extracellular signal regulated kinase
Flt1: VEGF receptor 1
HCASMC: human coronary artery smooth muscle cell
HEK293T: human embryonic kidney cell line
HRP: horseradish peroxidase
HUVEC: human umbilical vein endothelial cell
ICAM1/CD54: intercellular adhesion molecule-1
IFN-γ: interferon-gamma
IKK2: inhibitor of NF-κB kinase beta subunit
IL-1β: interleukin-1beta
isoQC: iso-glutaminyl cyclase
Jak: Janus kinase
KDR: VEGF receptor 2
MAPK: mitogen-activated protein kinase
MEK: MAPK/ERK kinase
NF-κB: nuclear factor kappa B
NTC: non-target control
p38: p38 mitogen-activated protein kinase
pGlu: pyroglutamate
PI3K: phosphatidylinositol-3 kinase
QC: glutaminyl cyclase
qPCR: quantitative PCR
QPCT: gene name of QC
QPCTL: gene name of isoQC
SMC: smooth muscle cell
Stat: signal transducer and activator of transcription
TNF-α: tumor necrosis factor-alpha
VCAM1: vascular cell adhesion molecule 1
VEGF: vacular endothelial growth factor
