# Supplementary material for: N-terminal pyroglutamate formation in CX3CL1 is essential for its full biologic activity
Source: Biosci Rep. 2017 Aug 24;37(4):BSR20170712. doi: 10.1042/BSR20170712 (PMC5634468; doi:10.1042/BSR20170712)

Supplemental information

Supplemental Table 1.

Pooled siRNAs consisted of 4 single siRNAs with the following sequences

siRNA	Thermo Scientific ON-TARGETplus SMARTpool (Dharmacon RNAi Technologie)	
**pooled QPCT-siRNA**	consist of	target sequence
QPCT_1	J-012474-05, QPCT	CUAUGGGUCUCGACACUUA
QPCT_2	J-012474-06, QPCT	GUACCGGUCUUUCUCAAAU
QPCT_3	J-012474-07, QPCT	CCCUUAAAGACUGUUUCAGA
QPCT_4	J-012474-08, QPCT	GGAACUUGCUCGUGCCUUA
**Non Target Control**	NTC	unknown
siRNA	Qiagen FlexiTube siRNA	
**pooled QPCT-siRNA**	consist of	target sequence
QPCT_5	Hs_QPCT_5	CAGGTGGTTCGAAAGACTTCA
QPCT_6	Hs_QPCT_7	TTCGAAAGACTTCAAGCAATT
QPCT_7	Hs_QPCT_8	CAGGTTCATTCATTCAACGAT
QPCT_8	Hs_QPCT_9	CTCTATGGGTCTCGACACTTA

Supplemental Table 2.

Primer sequences of the human genes

**Name**	**Forward**	**Reverse**
CCL2	GCCTCCAGCATGAAAGTCTC	CAGATCTCCTTGGCCACAAT
ICAM1/CD54	GGCTGGAGCTGTTTGAGAAC	TCACACTGACTGAGGCCTTG
CX3CL1	TCTGCCATCTGACTGTCCTG	CTGTGCTGTCTCGTCTCCAA
GAPDH	ACCCAGAAGACTGTGGATGG	TTCTAGACGGCAGGTCAGGT
YWHAZ	AGCAGGCTGAGCGATATGAT	TCTCAGCACCTTCCGTCTTT

Fig 1 Supplement

Conversion of Q1-CX3CL1 to pE1-CX3CL1 in human serum

At time point 0 min, the major peak corresponds to a mass of 8630, which is close to the theoretical molecular weight (8639) of Q1-CX3CL1. After incubating Q1-CX3CL1 with human serum for 30 min, the major peak corresponds to a mass of 8613, which is close to the theoretical molecular weight (8622) of pE1-CX3CL1. The doublet at 8286/8303 corresponds to a shortened peptide produced by limited proteolysis at the C-terminus (release of C-terminal RNG tripeptide). The C-terminal cleavage is independent of N-terminal cyclisation reaction.

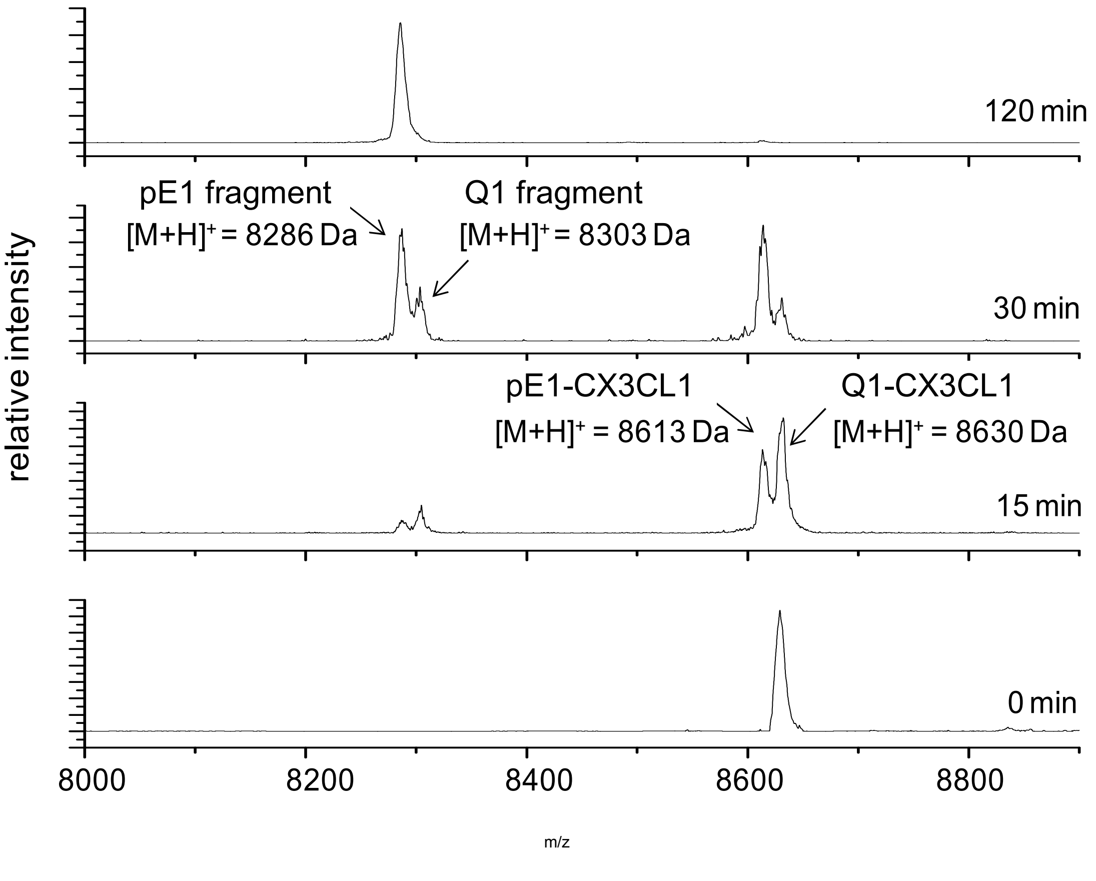

Supplement: Supplementary file 1 [file bsr20170712_Supp1.pdf]
